# Ly49E Expression on CD8αα-Expressing Intestinal Intraepithelial Lymphocytes Plays No Detectable Role in the Development and Progression of Experimentally Induced Inflammatory Bowel Diseases

**DOI:** 10.1371/journal.pone.0110015

**Published:** 2014-10-13

**Authors:** Aline Van Acker, Jessica Filtjens, Sophie Van Welden, Sylvie Taveirne, Els Van Ammel, Mandy Vanhees, Lindsey Devisscher, Tessa Kerre, Tom Taghon, Bart Vandekerckhove, Jean Plum, Georges Leclercq

**Affiliations:** 1 Department of Clinical Chemistry, Microbiology and Immunology, Ghent University, Ghent, Belgium; 2 Department of Gastroenterology, Ghent University, Ghent, Belgium; Karolinska Institutet, Sweden

## Abstract

The Ly49E NK receptor is a unique inhibitory receptor, presenting with a high degree of conservation among mouse strains and expression on both NK cells and intraepithelial-localised T cells. Amongst intraepithelial-localised T cells, the Ly49E receptor is abundantly expressed on CD8αα-expressing innate-like intestinal intraepithelial lymphocytes (iIELs), which contribute to front-line defense at the mucosal barrier. Inflammatory bowel diseases (IBDs), encompassing Crohn's disease and ulcerative colitis, have previously been suggested to have an autoreactive origin and to evolve from a dysbalance between regulatory and effector functions in the intestinal immune system. Here, we made use of Ly49E-deficient mice to characterize the role of Ly49E receptor expression on CD8αα-expressing iIELs in the development and progression of IBD. For this purpose we used the dextran sodium sulphate (DSS)- and trinitrobenzenesulfonic-acid (TNBS)-induced colitis models, and the TNF^ΔARE^ ileitis model. We show that Ly49E is expressed on a high proportion of CD8αα-positive iIELs, with higher expression in the colon as compared to the small intestine. However, Ly49E expression on small intestinal and colonic iIELs does not influence the development or progression of inflammatory bowel diseases.

## Introduction

Inflammatory bowel diseases (IBDs), encompassing Crohn's disease and ulcerative colitis, are chronic and relapsing disorders of the gastrointestinal tract [1]. Although the etiology of IBD is incompletely understood, inflammatory bowel disorders are believed to present in genetically predisposed individuals exposed to undefined microbial and environmental triggers. In this, IBD pathogenesis has been linked to deregulation of the fine homeostatic balance that exists between the mucosal immune system and commensal microbiota [2]–[5]. With the highest worldwide prevalence, European figures show an estimated 1 in 200 people affected by ulcerative colitis, and 1 in 300 affected by Crohn's disease [6], [7]. Current IBD treatment options include the administration of anti-inflammatory drugs, immunosuppressives and immunobiological agents [8], [9]. For unresponsive patients, surgical intervention may provide a temporary relieve from symptoms. However, specificity is lacking in these modes of treatment, and none are capable of inducing complete remission [8], [10]. Therefore, additional research is required to further elucidate IBD mechanisms and facilitate the development of specific and effective new therapies.

Intestinal intraepithelial lymphocytes (iIELs) are resident lymphocytes of the intestinal epithelium, and constitute one of the largest lymphocyte populations of the body [11]. In mice, five main subpopulations of iIELs have been identified: CD4, CD8αβ and CD8αα T cell receptor (TCR)αβ iIELs, and CD4/CD8 double-negative (DN) and CD8αα TCRγδ iIELs. TCRαβ CD4 and TCRαβ CD8αβ iIELs have been described as being ‘induced’ iIELs, whereas TCRαβ CD8αα, TCRγδ DN and TCRγδ CD8αα iIELs are also referred to as ‘natural’ iIELs [12]. More recently, Mucida *et al.* [13] have also described the existence of a sixth iIEL subpopulation, the TCRαβ CD4 CD8αα double-positive (DP) iIELs. Numerous reports indicate an important role for iIELs in maintaining mucosal homeostasis. In this context, iIELs have been implicated in the recognition of stress signals [14], repair of the intestinal epithelium [15]–[17], may function as memory cells [18], show cytotoxic activity [19], [20], and have autoreactive properties [21], [22]. In addition, iIELs are characterized by an ‘activated yet resting’ phenotype [20], suggesting the capacity to respond rapidly to *in vivo* stimuli and the need for tight regulation of iIEL effector function. However, exact mechanisms involved in iIEL regulatory and effector functions remain largely unknown.

Unique to iIELs is the large number of cells expressing the CD8αα homodimer [23]. Recently, our group showed that Ly49E, an inhibitory receptor, is abundantly expressed on CD8αα-expressing iIELs of the small intestine [24]. We demonstrated that iIELs expressing inhibitory Ly49 receptors, including Ly49E, are hyporesponsive to TCR-mediated stimulation [24]. Importantly, *in vitro* TCR-triggering results in upregulation of Ly49E receptor expression on iIELs [25]. Furthermore, we were able to show that the Ly49E receptor can be triggered by the non-MHC-related protein urokinase plasminogen activator (uPA). Interestingly, several studies have indicated a role for uPA in IBD, with increased levels of tissue uPA present in Crohn's disease and ulcerative colitis patients as compared to healthy controls [26], [27]. Thus, we hypothesized that Ly49E expression on CD8αα-expressing iIELs constitutes a novel mechanism through which iIEL function can be regulated. To test our hypothesis *in vivo*, we have generated Ly49E knockout (KO) mice on a C57BL/6 background. Here, we investigated a role for Ly49E expression on iIELs in the context of IBD development and progression, making use of the dextran sodium sulphate (DSS)- and trinitrobenzenesulfonic-acid (TNBS)- induced colitis models, and the TNF^ΔARE^ ileitis model. Our results indicate that Ly49E expression on both small intestinal and colonic CD8αα-expressing iIELs is abundant. However, abrogation of Ly49E expression on iIELs of the small intestine and colon does not influence development or progression of IBD.

## Materials and Methods

### Mice

The generation of Ly49E KO mice on a C57BL/6 background was outsourced to Ozgene (Bentley DC, WA, Australia), and the targeting strategy is explained elsewhere [28]. Heterozygous Ly49E^WT/KO^ mice were interbred to obtain homozygous Ly49E^WT/WT^ and Ly49E^KO/KO^ mice. TNF^ΔARE/WT^ mice were a kind gift from Dr. G. Kollias (Institute for Immunology, Biomedical Sciences Research Center “Alexander Fleming,” Attica, Greece). Heterozygous TNF^ΔARE/WT^ (Ly49E^WT/WT^) mice were bred to homozygous (TNF^WT/WT^) Ly49E^KO/KO^ mice in order to obtain TNF^ΔARE/WT^ Ly49E^WT/KO^ offspring. Male TNF^ΔARE/WT^ Ly49E^WT/KO^ and female TNF^WT/WT^ Ly49E^WT/KO^ offspring were bred to obtain homozygous TNF^ΔARE/WT^ Ly49E^WT/WT^ and TNF^ΔARE/WT^ Ly49E^KO/KO^ mice. All mice were housed and bred in our animal facility, and all animal experimentation was performed after approval and according to the guidelines of the Ethical Committee for Experimental Animals at the Faculty of Medicine and Health Sciences of Ghent University (Ghent, Belgium).

### Genotyping of mice

Genomic DNA was extracted from tail tissue samples, according to the REDExtract-N-Amp Tissue PCR Kit (Sigma-Aldrich, St. Louis, MO, USA). PCR amplification of genomic DNA for the TNF locus was carried out with the following primers: forward-5′CCTTCCTCACAGAGCCAGC-3′ and reverse-5′-AATTACGGTTAGGCTCCTGTTTCC-3′. Following agarose gel electrophoresis, TNF^ΔARE^ and wild-type (WT) alleles yield bands of 626 and 560 bp, respectively. PCR amplification of genomic DNA for the Ly49E locus was carried out making use of the following primers: for the WT allele, forward-5′-TCGCTTGGAATCTTCTGTTTC-3′ and reverse-5′TCCTCACCTGGACTGCAATC-3′; for the KO allele, forward-5′GGAATAATTGCTGTTACCATTAG in combination with reverse-5′TCCTCACCTGGACTGCAATC-3′. Ly49E WT and KO alleles yield bands of 1070 and 1200 bp, respectively.

### DSS-induced colitis

Colitis was induced in 8-week old mice by administering 4% (w/v) DSS (molecular weight 36,000–50,000, MP Biomedicals, Cleveland, USA) in drinking water and allowing mice to drink *ad libidum* for 7 days. Following 7 days of DSS treatment, drinking water containing DSS was replaced by normal drinking water and mice were allowed to recover. Control mice received normal drinking water only.

### Assessment of the disease activity index in DSS-induced colitis

The disease activity index (DAI) was scored as follows: weight loss, score 0–5 (0: <1% change in weight, 1: 1–5% change in weight, 2: 6–10% change in weight, 3: 11–15% change in weight, 4: 16–20% change in weight, 5: >20% change in weight), appearance, score 0–2 (0: healthy appearance, 1: unkempt fur coat, 2: arched back), fecal consistency, score 0–2 (0: normal, 1: soft pellets, 2: diarrhea) and fecal occult blood, score 0–5, using the ColoScreen Hemoccult test (Helena Laboratories, Texas, USA) (0: Hemoccult negative, 1: Hemoccult +, 2: Hemoccult ++, 3: Hemoccult +++, 4: Hemoccult ++++ or marked bleeding, 5: visible indications of rectal bleeding and prolapse). An individual score was assigned to each mouse on a daily basis.

### TNBS-induced colitis

Prior to administration of TNBS, mice were sedated through intraperitoneal injection with a mixture (2.5 µl/g body weight) of ketamine (50 mg/ml: 4 volumes) and xylazine (2%: 1 volume). For induction of TNBS-induced colitis, mice were rectally administered a solution (0.1 ml/mouse) of 2.5% (w/v) TNBS (Sigma-Aldrich) in 50% ethanol solution. Control mice received a solution (0.1 ml/mouse) of PBS in 50% ethanol solution. Mice were kept in a vertical position for 1 min to prevent rectal leakage.

### iIEL isolation

iIELs from 8-to-14 week old mice were isolated as follows. Briefly, intestines were removed and cleaned of mesenteric fat. Peyer's patches were excised, and intestines were rinsed with DPBS to clear intestines of fecal content. Subsequently, intestines were opened longitudinally, cut into 0.5-cm pieces, and transferred to a 50-ml conical tube. Intestines were incubated twice for 20 minutes at 37°C in Ca/Mg-free Hank's Balanced Sodium Solution (HBSS; Invitrogen, Carlstad, CA, USA) containing 5% FCS, 1 mM ethylenediaminetetraacetic acid (EDTA; Invitrogen) and 1 mM dithiotreitol (Sigma-Aldrich) at slow rotation. Cell suspensions were passed through a 40-µm cell strainer (Falcon, Becton Dickinson, Franklin Lakes, NJ, USA) and pelleted by centrifugation at 480 g. Pellets were resuspended in 44% Percoll (GE Healthcare, Buckinghamshire, UK) on an underlay of 67% Percoll, and centrifugated for 20°C at 2000 g. iIELs were collected from the 44%/67% Percoll interface, washed twice with phosphate buffered saline (PBS) for 5 minutes at 840 g, and resuspended in RPMI 1640 medium supplemented with 10% FCS, 100 U/ml penicillin, 100 µg/ml streptomycin, 2 mM glutamine, 1 mM sodium pyruvate, 100 µM non-essential amino acids (all Invitrogen) and 50 µM 2-mercaptoethanol (Sigma-Aldrich).

### Antibodies

mAbs used for staining of iIELs were as follows: anti-TCRβ (APC/Cy7-conjugated, clone H57.597), anti-CD4 (peridinin chlorophyll protein/Cy5.5-conjugated, clone GK1.5), anti-CD8β (peridinin chlorophyll protein/Cy5.5-conjugated, clone YTS156.7.7), all from BioLegend, San Diego, CA, USA. Anti-TCRδ (PE- or FITC-conjugated, clone GL3), anti-Ly49D (FITC-conjugated, clone 4E5), anti-Ly49C/I (PE-conjugated, clone 5E6), anti-NKG2D (PE-conjugated, clone CX5), anti-CD69 (biotin-conjugated, clone H1.2F3), were obtained from Becton Dickinson, Franklin Lakes, NJ, USA. Anti-CD8α (PE/Cy7-conjugated, clone 53–6.7) from eBioscience, San Diego, CA, USA. Anti-Ly49G2-producing hybridoma (clone 4D11) was from ATCC and antibody was biotin- or FITC-conjugated in-house. Anti-Ly49A (biotin-conjugated, clone JR9-318; kindly provided by Dr J. Roland (Paris, France)), anti-Ly49E/C (biotin- or FITC-conjugated, clone 4D12, made and labeled in-house) [29] and anti-Ly49E/F (FITC-conjugated, clone CM4; kindly provided by Dr C. G. Brooks (Newcastle on Tyne, UK) [30]. mAb 4D12 (Ly49C/E) in combination with mAb CM4 (Ly49E/F) were used to identify Ly49C-expressing cells (CM4^−^/4D12^+^), Ly49E-expressing cells (CM4^+^/4D12^+^) and Ly49F-expressing cells (CM4^+^/4D12^−^). In B6 (H-2^b^) mice, mAb 5E6 stains Ly49I, whereas Ly49C is very hard to detect [31]. Anti-Ly49H (biotin-conjugated, clone 3D10; kindly provided by Dr. W. Yokoyama, St.Louis, MO, USA)) and anti-NKG2A/C/E (FITC-conjugated, clone 3S9, generated and labeled in-house) [29].

Prior to staining, cells were blocked with anti-FcγRII/III (unconjugated, clone 2.4G2, kindly provided by Dr J. Unkeless, Mount Sinai School of Medicine, New York, USA). Propidium iodide was used to discriminate live and dead cells. Flow cytometry was performed using a BD LSRII flow cytometer, and samples were analysed with FACSDiva Version 6.1.2 software (BD Biosciences).

### Histology

For histological analysis, tissue sections of the distal ileum and distal colon were fixed in 4% formaldehyde solution (VWR, Radnor, Pennsylvania, USA) and embedded in paraffin. 5 µm paraffin-embedded sections were stained with hematoxylin and eosin (Sigma-Aldrich). Intestinal inflammation was scored blindly by two independent observers using a validated scoring system, as previously described [32].

### Statistics

Statistical analysis was carried out using PASW Statistics 22 Software (SPSS, Chicago, IL, USA). Data was analysed using the non-parametric two-tailed Mann-Whitney U-test or ANOVA, as indicated. A *P* value ≤0.05 was considered statistically significant.

## Results

### Ly49E KO mice have normal iIEL population frequencies and NK receptor expression

**Figure 1:**
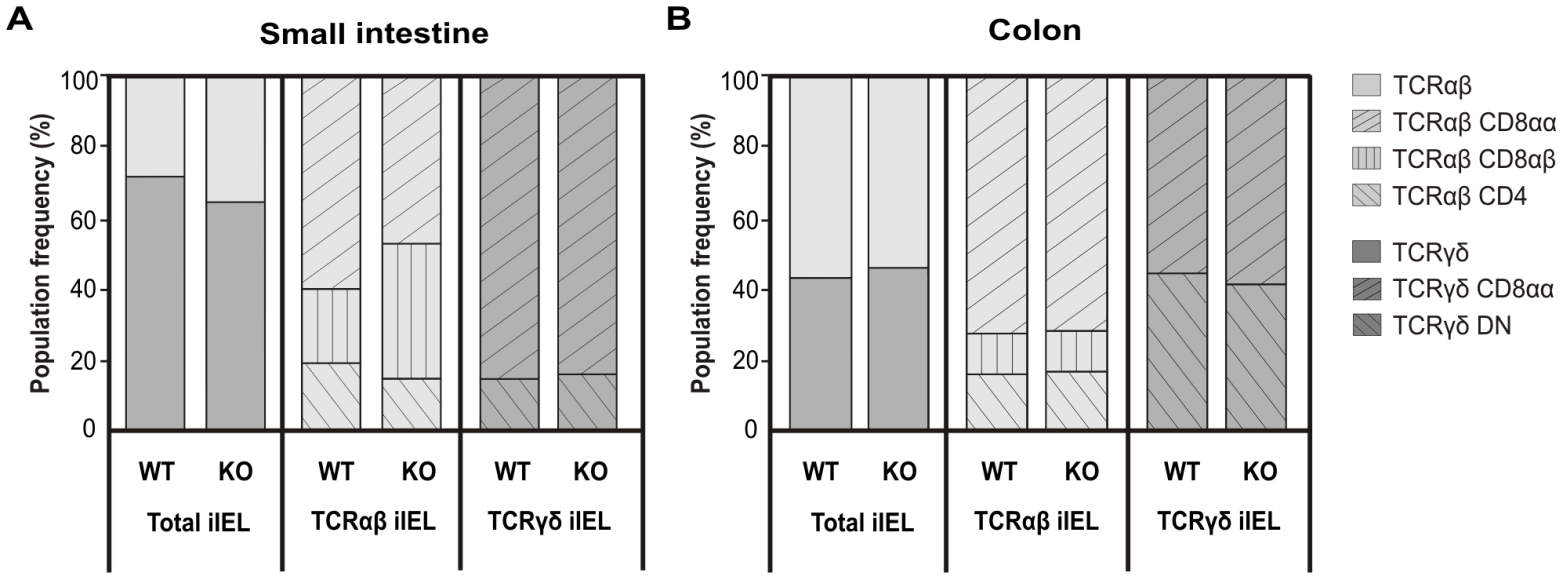
iIEL subpopulation frequencies in the small intestine and colon of Ly49E WT versus Ly49E KO mice. A) iIEL subpopulation frequencies in the small intestine of Ly49E WT versus Ly49E KO mice. TCRαβ and TCRγδ iIEL population frequencies are shown as a percentage of the total iIELs present in the small intestine and colon of Ly49E WT versus Ly49E KO mice. TCRαβ CD4, TCRαβ CD8αβ and TCRαβ CD8αα iIEL subpopulation frequencies are shown as a percentage of the total TCRαβ iIELs. TCRγδ DN and TCRγδ CD8αα iIEL subpopulation frequencies are shown as a percentage of the total TCRγδ iIELs. B) iIEL subpopulation frequencies in the colon of Ly49E WT versus Ly49E KO mice. Representation of iIEL subpopulation frequencies is the same as for Fig. 1A. iIEL subpopulation frequencies are shown as mean values (n = 5, small intestine; n = 5, colon, where each value is derived from a pool of 3 mice).

**Figure 2:**
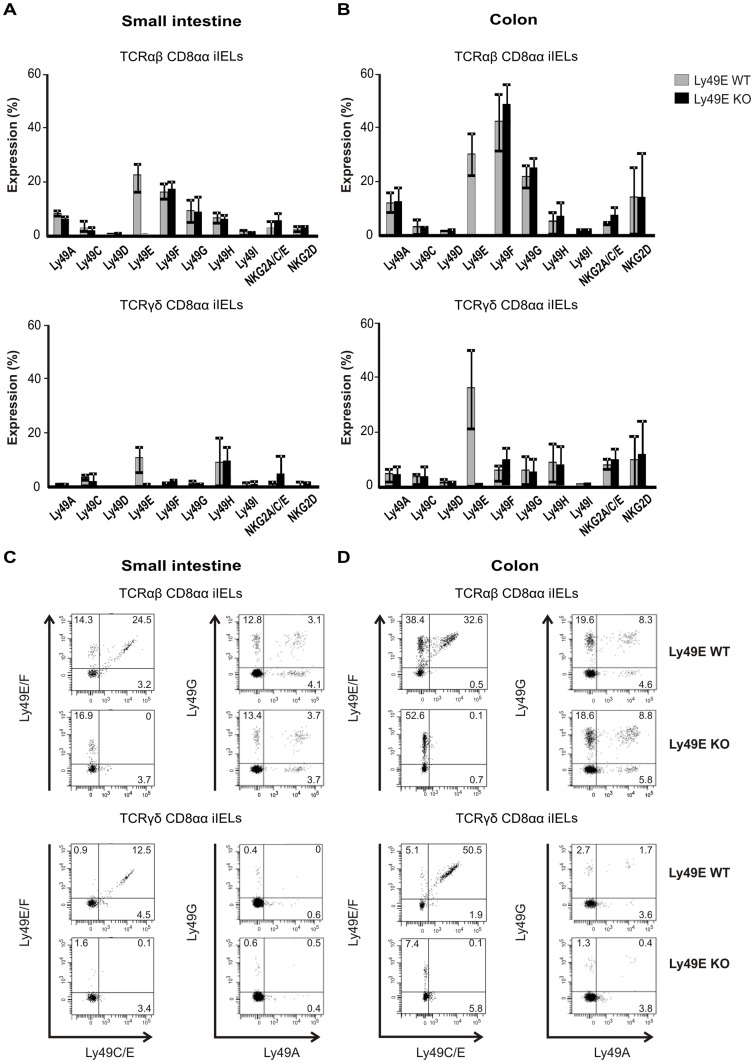
iIEL NK receptor expression in the small intestine and colon of Ly49E WT versus Ly49E KO mice. A) NK receptor expression on TCRαβ CD8αα iIELs and TCRγδ CD8αα iIELs in the small intestine of Ly49E WT versus Ly49E KO mice. B) NK receptor expression on TCRαβ CD8αα iIELs and TCRγδ CD8αα iIELs in the colon of Ly49E WT versus Ly49E KO mice. C) Co-expression of Ly49 receptors on the iIEL surface of TCRαβ CD8αα iIELs and TCRγδ CD8αα iIELs in the small intestine of Ly49E WT versus Ly49E KO mice. Numbers indicate the percentage of cells expressing or co-expressing the indicated Ly49 receptors, and are representative of 3 mice. D) Co-expression of Ly49 receptors on the iIEL surface of TCRαβ CD8αα iIELs and TCRγδ CD8αα iIELs in the colon of Ly49E WT versus Ly49E KO mice. Numbers indicate the percentage of cells expressing or co-expressing the specific Ly49 receptors, and are representative of 3 mice. iIEL NK receptor expression is presented as the mean ±SD (n = 5, small intestine; n = 5, colon, where each value represents the mean of a pool of 3 mice).

As the function of Ly49E expression on iIELs of the intestine is currently unknown, we initially sought to clarify whether Ly49E expression on iIELs affects differentiation of intestinal iIELs. In this respect, we found that total numbers of iIELs present in the small intestine of Ly49E WT and Ly49E KO mice (8.68×10^6^±2.50 vs. 6.60×10^6^±1.15 iIELs, respectively) and colon of Ly49E WT and Ly49E KO mice (0.32×10^6^±0.10 vs. 0.46×10^6^±0.20) were similar. Furthermore, iIEL subpopulation frequencies, and the total number of cells in each iIEL subpopulation, did not differ significantly between Ly49E WT and Ly49E KO mice (Fig. 1A and 1B, and data not shown). We and others have previously shown that several NK receptors are expressed on the surface of small intestinal iIELs. In particular, Ly49E is expressed on a higher proportion of CD8αα-positive iIELs than other Ly49 receptors [24], [33]. Here, we show that several NK receptors are also expressed on the surface of colonic CD8αα-positive iIELs. As for small intestinal iIEL expression, a high proportion of colonic iIELs express the Ly49E receptor. Surprisingly, the frequency of Ly49E-expressing colonic TCRγδ CD8αα-positive iIELs exceeds the frequency of Ly49E-expressing TCRγδ CD8αα-positive iIELs in the small intestine, whereas we found the proportion of Ly49E-expressing TCRαβ CD8αα-positive iIELs in the small intestine and colon to be comparable. As expected, Ly49E expression is absent on iIELs of Ly49E KO mice. Small intestinal and colonic iIEL NK receptor expression was unchanged between Ly49E WT and Ly49E KO mice (Fig. 2A and 2B). Furthermore, co-expression of Ly49 receptors on the iIEL surface was unaltered between Ly49E WT and Ly49E KO mice (Fig. 2C and 2D). Ly49E expression on CD8αα-negative iIELs was low or absent (data not shown).

### Ly49E expression on CD8αα-expressing iIELs of the colon does not influence DSS-induced colitis

**Figure 3:**
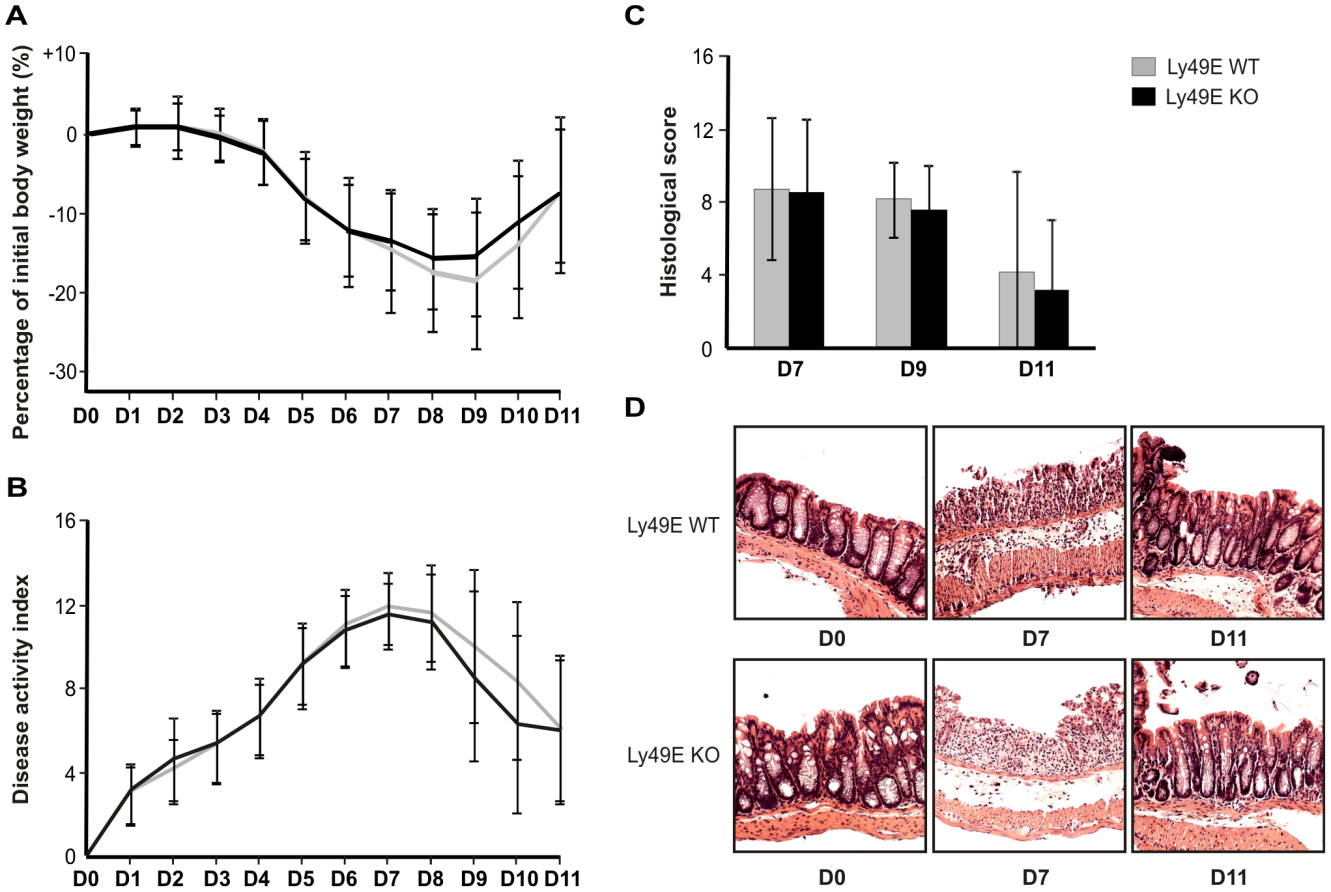
Clinical symptoms of DSS-induced colitis in Ly49E WT versus Ly49E KO mice. Colitis was induced in Ly49E WT and Ly49E KO mice by administration of DSS in drinking water for 7 days. Thereafter, mice received normal drinking water. Ly49E WT and Ly49E KO mice were scored and compared at the indicated days. A) Relative weight loss (mean ±SD; n = 71). The reference weight was taken as the weight on day 0, at the start of the experiment. B) Disease activity index (mean ±SD; n = 71). C) Colon histological score (mean ±SD; n = 6 on day 7, n = 5 on day 9 and n = 10 on day 11). D) Representative hematoxylin/eosin-stained paraffin sections of the distal colon.

**Figure 4:**
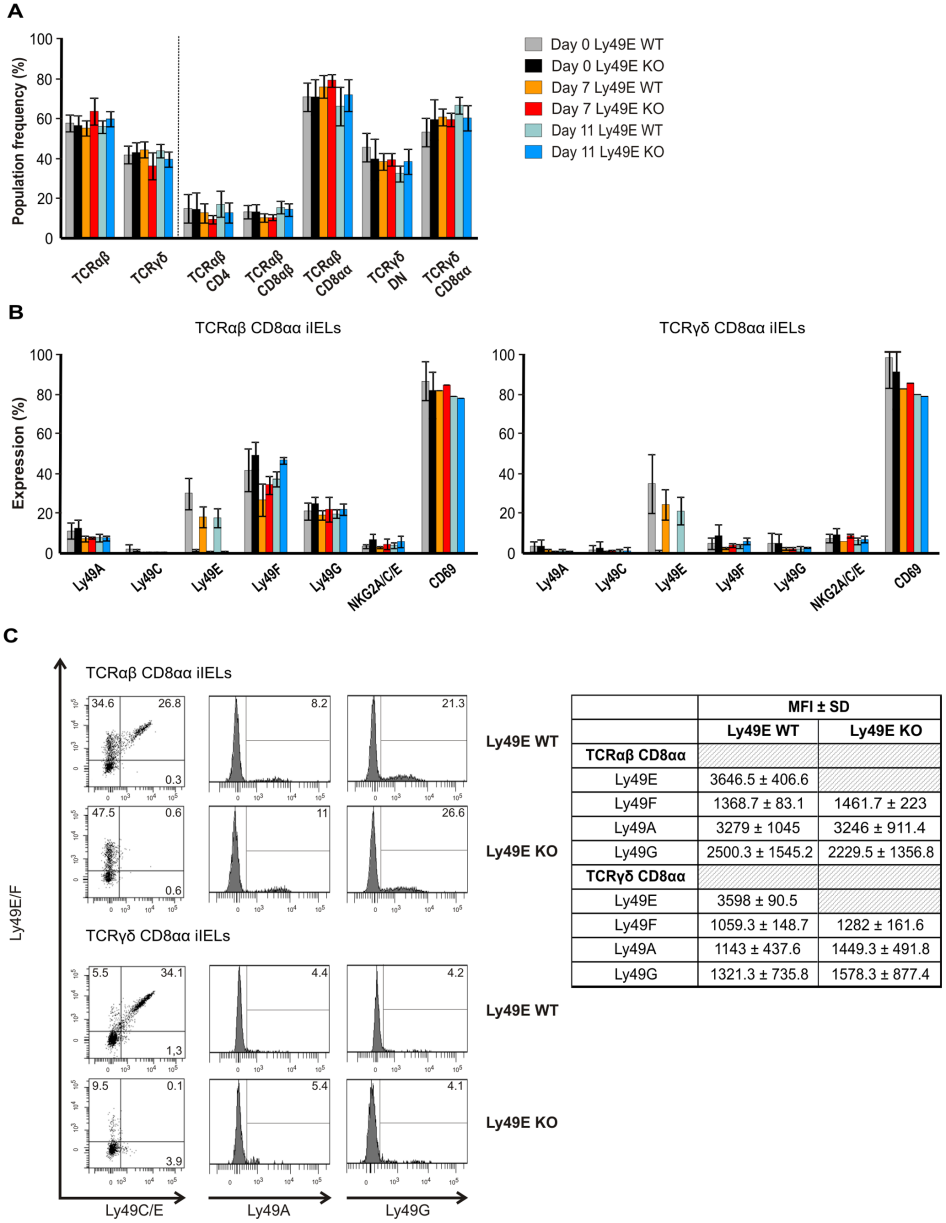
iIEL NK receptor expression upon DSS-induced colitis in Ly49E WT versus Ly49E KO mice. A) Colon iIEL subpopulation frequencies (mean ±SD; n = 5 on day 0, n = 3 on day 7 and n = 4 on day 11, where each value is derived from a pool of 3 mice). TCRαβ CD4, TCRαβ CD8αβ and TCRαβ CD8αα iIEL subpopulation frequencies are shown as a percentage of the total TCRαβ iIELs. TCRγδ DN and TCRγδ CD8αα iIEL subpopulation frequencies are shown as a percentage of the total TCRγδ iIEL. B) Colon iIEL NK receptor and activation marker expression on TCRαβ CD8αα iIELs and TCRγδ CD8αα iIELs (mean ±SD; n = 5 on day 0, n = 3 on day 7 and n = 4 on day 11, where each value represents the mean of a pool of 3 mice). C) Colon iIEL NK receptor expression on TCRαβ CD8αα iIELs and TCRγδ CD8αα iIELs on day 7 following induction of DSS-induced colitis. Numbers indicate the percentage of cells expressing the indicated Ly49 receptors, and are each representative of 5 mice. Representative data are shown on the left. Mean fluorence intensity (MFI) values for each receptor are shown on the right.

**Figure 5:**
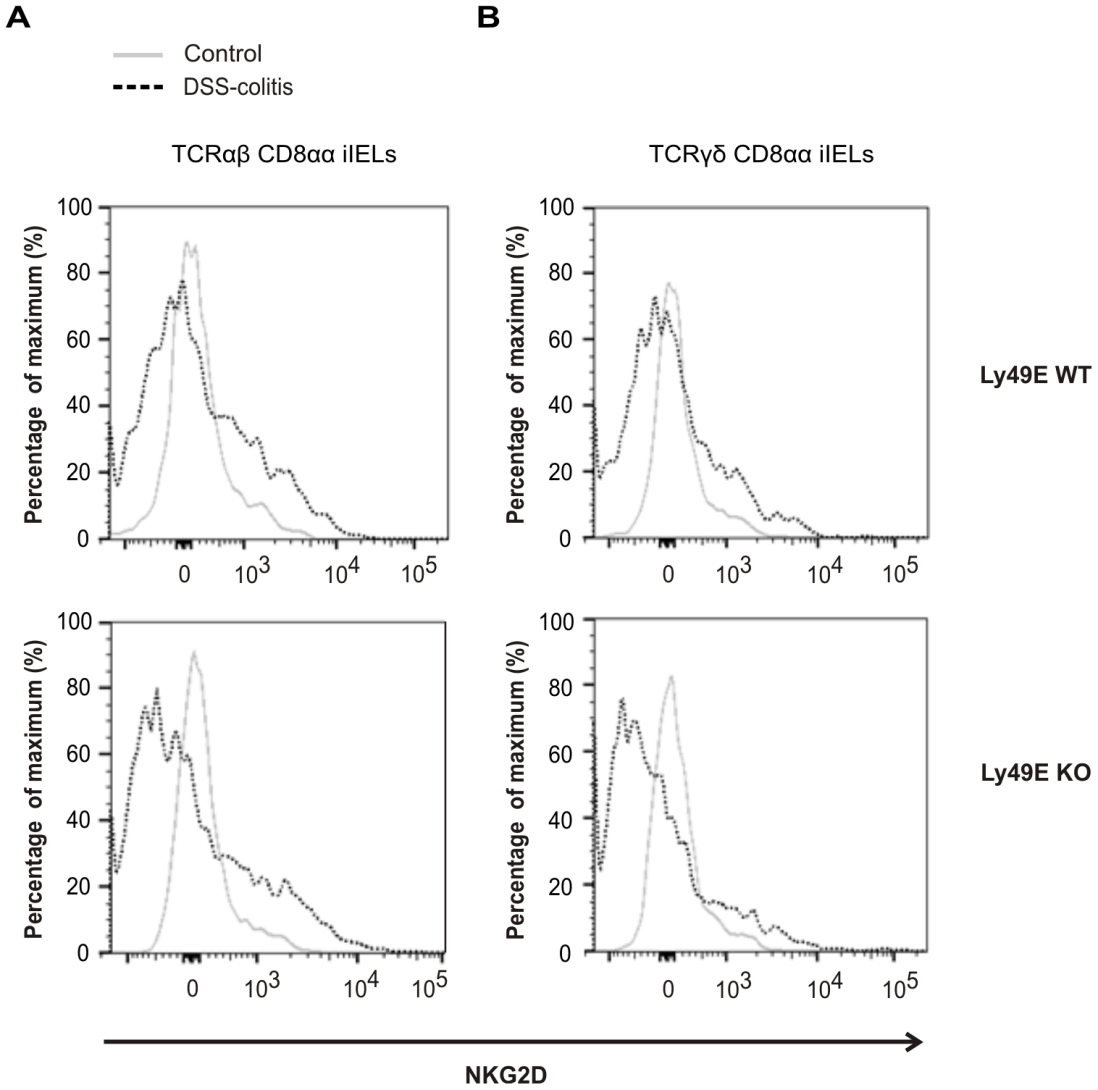
iIEL NKG2D expression upon DSS-induced colitis in Ly49E WT and Ly49E KO mice. Colitis was induced in Ly49E WT and Ly49E KO littermate mice by administration of DSS in drinking water for 7 days. A) NKG2D expression on TCRαβ CD8αα iIELs. B) NKG2D expression on TCRγδ CD8αα iIELs. N = 2 for control mice, n = 3 for DSS-treated mice.

To study a possible role for Ly49E expression on colonic CD8αα-expressing iIELs in the context of colitis, we performed DSS-induced colitis with Ly49E WT and Ly49E KO mice. DSS was administered to mice in drinking water for a period of 7 days, after which DSS-containing water was replaced by normal drinking water. Mice were analysed on a daily basis for a total of 11 days. As shown in Fig. 3A, we observed no difference in relative weight loss between Ly49E WT and Ly49E KO mice. Additionally, we analysed the DAI on a daily basis. Here, we observed no significant difference between the DAI of Ly49E WT and Ly49E KO mice between days 0–11 (p≥0.35; Mann-Whitney test) (Fig. 3B). This was corroborated by histological scores of distal colon formalin fixed paraffin-embedded (FFPE-) sections obtained from Ly49E WT and Ly49E KO mice at days 7, 9 and 11 following colitis induction, which indicate a similar degree of inflammation for Ly49E WT and Ly49E KO mice (Fig. 3C and 3D). Studying a role for Ly49E expression on colonic iIELs, we analysed iIEL subpopulation frequencies and iIEL NK receptor and activation marker expression at days 0, 7 and 11 of DSS-induced colitis. As illustrated, iIEL subpopulation frequencies and iIEL NK receptor and activation marker expression were unchanged on days 7 and 11 as compared to untreated mice on day 0, and were unchanged between Ly49E WT and Ly49E KO mice (Fig. 4A, 4B and 4C). Hall *et al.* [34] recently reported an increased frequency of NKG2D expression on NK cells in DSS-induced colitis. As iIELs have a number of innate-like properties and are crucial to front-line defense of the intestinal mucosal barrier [12], [18]–[22], we examined whether iIELs upregulate NKG2D expression upon DSS-induced colitis. Using littermate DSS-treated and control mice, we observe a trend towards upregulation of NKG2D expression on TCRαβ CD8αα-positive iIELs and TCRγδ CD8αα-positive iIELs in DSS-induced colitis, but this was not significant (p≥0.083; Mann-Whitney test). NKG2D expression between Ly49E WT and Ly49E KO DSS-treated mice was comparable (Fig. 5A and 5B). Thus, Ly49E expression on colonic CD8αα-expressing iIELs does not appear to influence DSS-induced colitis development or progression.

### Ly49E expression on CD8αα-expressing iIELs of the colon does not affect TNBS-induced colitis

**Figure 6:**
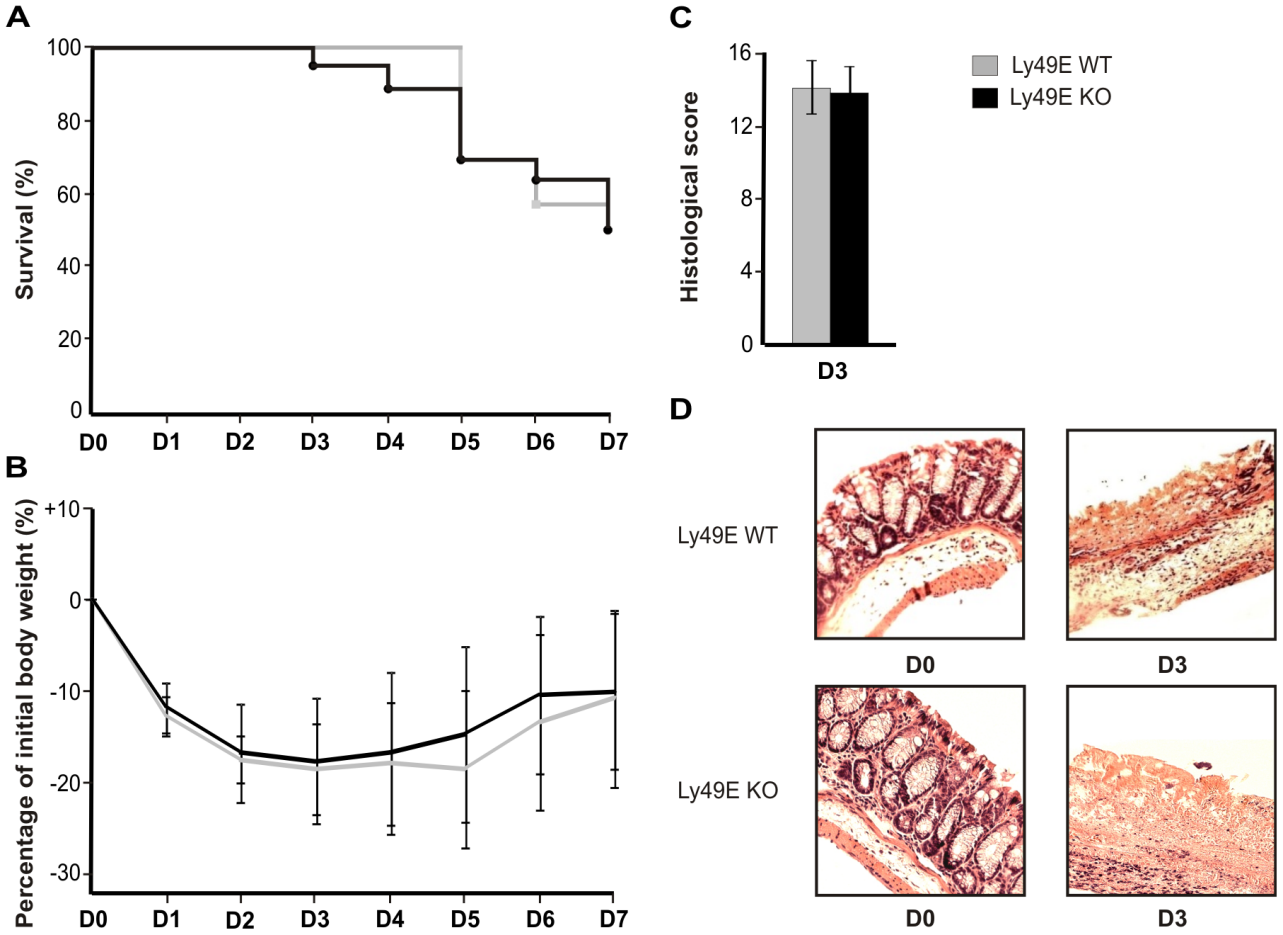
TNBS-induced colitis in Ly49E WT versus Ly49E KO mice. Colitis was induced in Ly49E WT and Ly49E KO mice by intra-rectal administration of TNBS. Mice were analysed at the indicated days. A) Survival (n = 16). B) Relative weight loss (mean ±SD; n = 16). The reference weight was taken as the weight on day 0, at the start of the experiment. C) Colon histological score (mean ±SD; n = 5). D) Representative hematoxylin/eosin- stained paraffin sections of the distal colon.

A second frequently used model in the study of mucosal immunity is that of TNBS-induced colitis. Here, we show that colitis induction in both Ly49E WT and Ly49E KO occurred rapidly, with the first cases of mortality noted at 5 and 3 days post-treatment for Ly49E WT and Ly49E KO mice, respectively. Seven days following TNBS-colitis induction, 50% of mice had died in both Ly49E WT and Ly49E KO-treated groups, and the decision was made to terminate treatment and euthanize the remaining mice (Fig. 6A). Maximum weight loss for Ly49E WT and Ly49E KO mice occurred at day 5 and day 3, respectively, following the start of treatment, and weight loss differences between Ly49E WT and KO mice were not significant (Mann-Whitney test) on all days analysed (Fig. 6B). FFPE-sections of the distal colon of Ly49E WT and Ly49E KO mice were obtained 72 h following induction of colitis, and scored as described. As shown in Fig. 6C and 6D, colonic inflammation in the colon of both Ly49E WT and Ly49E KO treated mice was comparable in severity. A role for Ly49E expression on colonic CD8αα-expressing iIELs was investigated by comparing iIEL numbers, iIEL subpopulation frequencies and iIEL NK receptor expression of Ly49E WT and Ly49E KO-treated mice 72 h after treatment start. Here, we found the total number of iIELs, iIEL subpopulation frequencies and iIEL NK receptor expression to be unchanged between Ly49E WT and Ly49E KO mice 72 h after treatment initiation as compared to healthy control mice (data not shown). Therefore, we conclude that Ly49E expression on colonic CD8αα-expressing iIELs does not alter TNBS-induced colitis development or progression.

### TNF^ΔARE/WT^ Ly49E^WT/WT^ and TNF^ΔARE/WT^ Ly49E^KO/KO^ mice display similar ileitis development and progression

**Figure 7:**
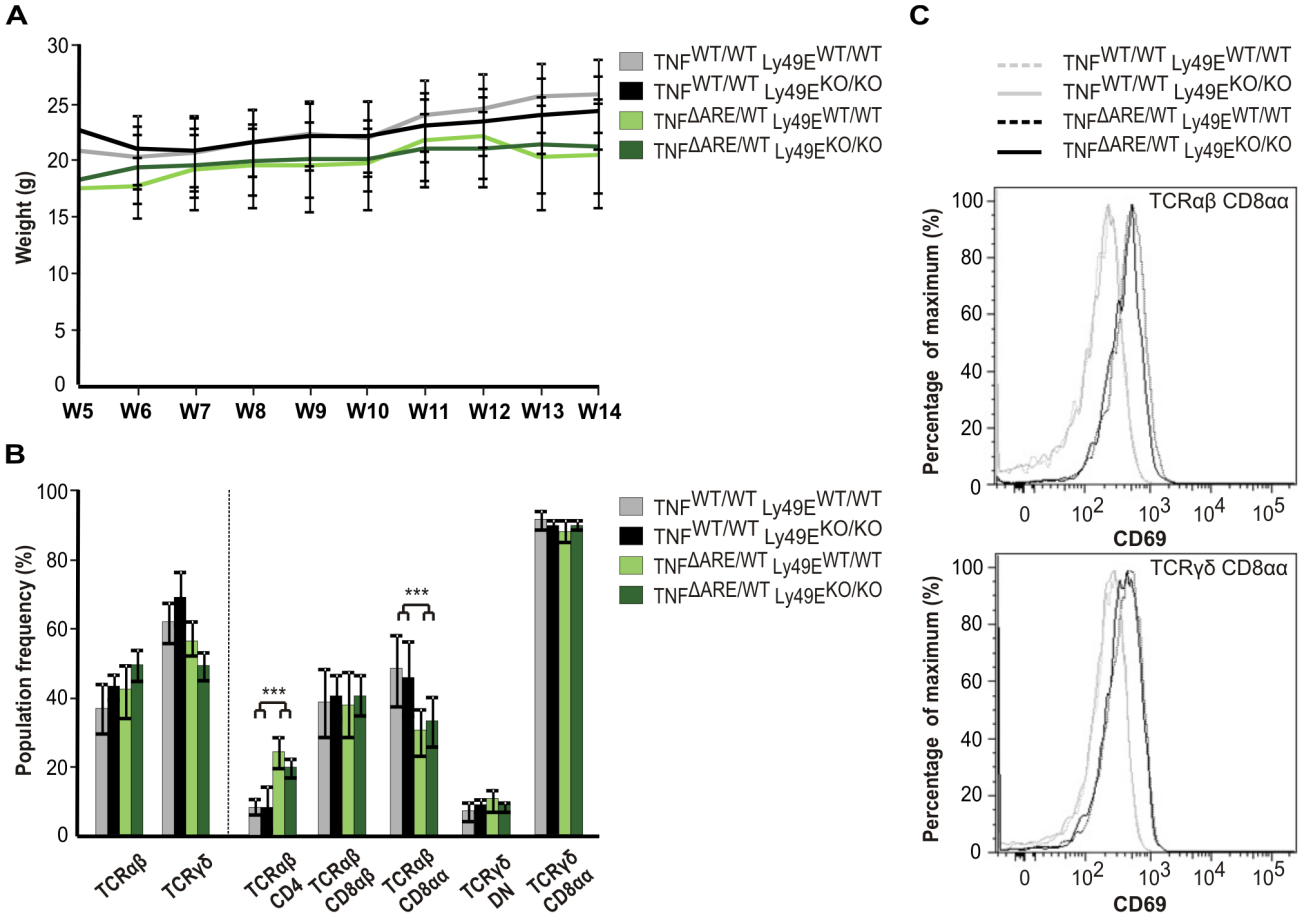
TNF^ΔARE^-induced ileitis on an Ly49E WT versus Ly49E KO background. A) Weight progression of TNF^ΔARE/WT^ Ly49E^WT/WT^ and TNF^ΔARE/WT^ Ly49E^KO/KO^ mice as compared to TNF^WT/WT^ Ly49E WT and Ly49E KO mice (mean ±SD; n = 30 (TNF^ΔARE/WT^ Ly49E^WT/WT^), n = 22 (TNF^ΔARE/WT^ Ly49E^KO/KO^), n = 31 (TNF^WT/WT^ Ly49E WT) and n = 21 (TNF^WT/WT^ Ly49E KO)). Weight was monitored weekly from the age of genotyping (5 weeks of age) up to 14 weeks of age. B) Small intestinal iIEL subpopulation frequencies in TNF^ΔARE/WT^ Ly49E^WT/WT^ and TNF^ΔARE/WT^ Ly49E^KO/KO^ mice as compared to TNF^WT/WT^ Ly49E WT and Ly49E KO mice (mean ±SD; n = 6). TCRαβ CD4, TCRαβ CD8αβ and TCRαβ CD8αα iIEL subpopulation frequencies are shown as a percentage of the total TCRαβ iIELs. TCRγδ DN and TCRγδ CD8αα iIEL subpopulation frequencies are shown as a percentage of the total TCRγδ iIEL. C) CD69 expression on small intestinal TCRαβ CD8αα iIELs and TCRγδ CD8αα iIELs of TNF^ΔARE/WT^ Ly49E^WT/WT^ and TNF^ΔARE/WT^ Ly49E^KO/KO^ mice as compared to TNF^WT/WT^ Ly49E WT and Ly49E KO mice (mean ±SD; n = 6).

To study the role of Ly49E expression on small intestinal iIELs in the context of ileitis development and progression, we employed the TNF^ΔARE^ model of ileitis. Harbouring a deletion of the TNF 3′ AU-rich elements, TNF^ΔARE/WT^ mice develop spontaneous ileitis that highly resembles human Crohn's disease [35], [36]. Here, we bred Ly49E WT and Ly49E KO mice to heterozygous TNF^ΔARE/WT^ mice in two rounds, generating TNF^ΔARE/WT^ Ly49E^WT/WT^ and TNF^ΔARE/WT^ Ly49E^KO/KO^ offspring. Monitoring weight progression of TNF^ΔARE/WT^ Ly49E^WT/WT^ and TNF^ΔARE/WT^ Ly49E^KO/KO^ mice, and their littermate controls, we noted decreased weights for TNF^ΔARE/WT^ mice as compared to TNF^WT/WT^ mice, as expected. Furthermore, we observed no significant difference in weight progression of TNF ^ΔARE/WT^ Ly49E^WT/WT^ versus TNF^ΔARE/WT^ Ly49E^KO/KO^ mice between 5 and 14 weeks of age (Fig. 7A). Subsequently, we analysed iIEL numbers, subpopulation frequencies, NK receptor and activation marker expression in TNF^ΔARE/WT^ Ly49E^WT/WT^ and TNF^ΔARE/WT^ Ly49E^KO/KO^ mice and their littermate controls at the age of 10 weeks, by which time ileitis is fully developed. Total numbers of iIELs isolated from the small intestine of TNF^ΔARE/WT^ Ly49E^WT/WT^ and TNF^ΔARE/WT^ Ly49E^KO/KO^ mice were similar (8.57×10^6^±2.73 vs. 9.57×10^6^±3.39 iIELs, respectively). Studying iIEL subpopulation frequencies, we noted a significant increase in TCRαβ CD4 iIELs (p≤0.001; ANOVA), and a significant decrease in TCRαβ CD8αα iIELs (p≤0.001; ANOVA), in the intestines of TNF^ΔARE/WT^ Ly49E^WT/WT^ and TNF^ΔARE/WT^ Ly49E^KO/KO^ mice as compared to their littermate controls. However, no differences were observed in iIEL subpopulation frequencies between TNF^ΔARE/WT^ Ly49E^WT/WT^ and TNF^ΔARE/WT^ Ly49E^KO/KO^ mice (Fig. 7B). Similarly, we noted no significant differences in the NK receptor expression of iIELs from TNF^ΔARE/WT^ Ly49E^WT/WT^ and TNF^ΔARE/WT^ Ly49E^KO/KO^ mice (data not shown). To confirm that iIELs were activated in this TNF^ΔARE^ ileitis model, we stained iIELs for expression of the activation marker CD69. Small intestinal iIELs from TNF^ΔARE/WT^ Ly49E^WT/WT^ and TNF^ΔARE/WT^ Ly49E^KO/KO^ mice expressed significantly higher CD69 levels as compared to their littermate controls for both TCRαβ CD8αα and TCRγδ CD8αα iIELs (p≤0.001; ANOVA). However, CD69 expression levels were comparable between TNF^ΔARE/WT^ Ly49E^WT/WT^ and TNF^ΔARE/WT^ Ly49E^KO/KO^ mice, and between control Ly49E WT and Ly49E KO mice (Fig. 7C). All experiments were repeated with mice at 14 weeks of age, and similar results were obtained (data not shown). Together, this data suggests that Ly49E expression on small intestinal iIELs does not affect ileitis development or progression.

## Discussion

In this paper we show that the Ly49E NK receptor is abundantly expressed on CD8αα-expressing iIELs of the small intestine as well as the colon. Herein, the frequency of Ly49E-expressing colonic CD8αα-positive iIELs is greater than the frequency of Ly49E-expressing small intestinal CD8αα-positive iIELs. Because of the relatively high Ly49E expression on iIELs, we initially sought to investigate whether Ly49E expression influences iIEL differentiation or the iIEL NK receptor expression profile. In this regard, we show that Ly49E KO mice have unchanged iIEL subpopulation frequencies and a similar NK receptor expression profile to Ly49E WT. Thus, we show that Ly49E expression on iIELs does not affect the development or NK receptor expression profile of basal resting iIELs.

Upon iIEL activation, a number of reports have demonstrated a function for these cells in regulating the development and progression of ulcerative colitis. Recently, Meehan *et al*. [37] illustrated that interaction of CD100 on γδ-iIELs with plexin B2 on epithelial cells is required for keratinocyte-growth factor (KGF-1)-mediated epithelial repair following DSS-induced colitis. A role for iIELs in TNBS-induced colitis has been shown by Inagaki-Ohara *et al*. [38], who demonstrated that γδ T cell-deficient (Cδ^−/−^) mice show high susceptibility to TNBS-induced colitis at young age, and that subsequent transfer of γδ IELs to Cδ^−/−^ mice ameliorates TNBS-induced colitis. Roselli *et al.* [39] corroborated this result by illustrating that a probiotic-induced increase in γδ-positive iIELs suppresses TNBS-induced colitis development in treated mice. Most recently, Jiang *et al*. [40] were able to show that adoptive transfer of iIELs to NOD2^−/−^ mice, harboring a strongly reduced number of iIELs, significantly reduced the susceptibility to TNBS-induced colitis. Together, these reports suggest an important role for iIELs in the regulation and prevention of ulcerative colitis.

Alongside, NK receptor expression has been implicated in colitis development. Hall *et al*. [34] showed that NKG2A expression on NK cells protected mice from DSS-induced colitis development, where NK cells downregulated reactive oxygen species and cytokine production by activated neutrophils through direct cell-to-cell contact involving the NK cell inhibitory receptor NKG2A. Additional data from human studies supports a possible role of NK receptor expression in ulcerative colitis development and progression. In this context, the frequency of KIR2DL1 and KIR2DL3 genotypes was shown to be lower in ulcerative colitis patients as compared to control individuals [41]–[43]. Moreover, it was shown that KIR2DL1/HLA-C2 interaction negatively correlates with IBD development [44]. Inversely, KIR2DL2 and KIR2DS2 expression was linked to an increased incidence of ulcerative colitis [45]. Interestingly, several studies have suggested KIR receptors to be the human equivalent of Ly49 receptors in mice [46]–[48]. Taken together, these studies illustrate that NK receptor expression on colonic NK cells and some T cell subsets may influence the course of colitis development. However, to our knowledge, a role for NK receptor expression on iIELs, in the context of ulcerative colitis, had not previously been investigated.

Recently, our group demonstrated that expression of inhibitory Ly49 receptors on iIELs promotes hyporesponsiveness of these cells [24], and that *in vitro* TCR-triggering results in upregulation of Ly49E receptor expression on iIELs, indicating a negative feedback loop [25]. Thus, we hypothesized that Ly49E expression on colonic CD8αα-expressing iIELs might provide a new mechanism through which iIEL function can be regulated in colitis development and progression. Here, we show that Ly49E expression on colonic CD8αα-expressing iIELs does not influence the development or progression of DSS-induced colitis and TNBS-induced colitis. Relative weight progression, disease activity index scores, and histological scoring of FFPE-embedded colon sections showed no significant difference in DSS-induced colitis progression between Ly49E WT and Ly49E KO mice. Furthermore, iIEL numbers, iIEL subpopulation frequencies and iIEL phenotype were unaltered throughout DSS-induced colitis progression in Ly49E WT versus Ly49E KO mice. Similarly, survival rates, relative weight progression and iIEL kinetics were not statistically significant in TNBS-induced colitis between Ly49E WT and KO mice.

A role for iIELs in ileitis, as modeled by the mouse TNF^ΔARE^ model, has been reported by Apostolaki *et al.* [49], who showed that intestinal inflammation in TNF^ΔARE/WT^ mice is associated with a reduced presence of CD8αα-expressing iIELs. As in ulcerative colitis, human data studies show a negative influence of KIR2DL2/KIR2DL3 in Crohn's disease development and a protective effect for the KIR2DL1/HLA-C2 interaction [41]–[44]. Here, we crossed Ly49E WT and Ly49E KO mice to heterozygous TNF^ΔARE/WT^ mice in two rounds, generating TNF^ΔARE/WT^ Ly49E^WT/WT^ and TNF^ΔARE/WT^ Ly49E^KO/KO^ offspring for the study of the role of Ly49E expression on iIELs in ileitis development and progression. Our results show that TNF^ΔARE/WT^ Ly49E^WT/WT^ and TNF^ΔARE/WT^ Ly49E^KO/KO^ mice display with similar ileitis disease kinetics. Concurrent with results from Apostolaki *et al.* [49], we observe a significant decrease of TCRαβ CD8αα iIELs in the small intestine of TNF^ΔARE/WT^ mice compared to their littermate controls. Alongside, we note a significant increase of TCRαβ CD4 iIELs in TNF^ΔARE/WT^ mice compared to their littermate controls. Furthermore, we show activation of TNF^ΔARE/WT^ small intestinal iIELs in ileitis, with significantly higher CD69 expression in TNF^ΔARE/WT^ mice as compared to littermate controls. However, we observe no significant differences in iIEL numbers, subpopulation frequencies, NK receptor expression or activation marker expression between TNF^ΔARE/WT^ Ly49E^WT/WT^ and TNF^ΔARE/WT^ Ly49E^KO/KO^ mice, illustrating that Ly49E expression on small intestinal CD8αα-expressing iIELs does not influence ileitis development or progression.

Conclusively, we report that Ly49E expression is abundant on iIELs of the small intestine and colon. In this, Ly49E is expressed on a high proportion of CD8αα-expressing iIELs. However, Ly49E expression on CD8αα-expressing iIELs does not influence the development or progression of inflammatory bowel diseases.

## References

[1] Dharmani P, Leung P, Chadee K (2011) Tumor necrosis factor-alpha and Muc2 mucin play major roles in disease onset and progression in dextran sodium sulphate-induced colitis. PLoS One 6: e25058. 21949848 10.1371/journal.pone.0025058 PMC3176316

[2] Baumgart DC, Carding SR (2007) Inflammatory bowel disease: cause and immunobiology. Lancet 369: 1627–1640. 17499605 10.1016/S0140-6736(07)60750-8

[3] Dharmani P, Chadee K (2008) Biologic therapies against inflammatory bowel disease: a dysregulated immune system and the cross talk with gastrointestinal mucosa hold the key. Curr Mol Pharmacol 1: 195–212. 20021434 10.2174/1874467210801030195

[4] Khor B, Gardet A, Xavier RJ (2011) Genetics and pathogenesis of inflammatory bowel disease. Nature 474: 307–317. 21677747 10.1038/nature10209 PMC3204665

[5] Henderson P, van Limbergen JE, Schwarze J, Wilson DC (2011) Function of the intestinal epithelium and its dysregulation in inflammatory bowel disease. Inflamm Bowel Dis 17: 382–395. 20645321 10.1002/ibd.21379

[6] Molodecky NA, Soon IS, Rabi DM, Ghali WA, Ferris M, et al. (2012) Increasing incidence and prevalence of the inflammatory bowel diseases with time, based on systematic review. Gastroenterology 142: : 46–54 e42; quiz e30. 10.1053/j.gastro.2011.10.001 22001864

[7] Perse M, Cerar A (2012) Dextran sodium sulphate colitis mouse model: traps and tricks. J Biomed Biotechnol 2012: 718617. 22665990 10.1155/2012/718617 PMC3361365

[8] Baumgart DC, Sandborn WJ (2007) Inflammatory bowel disease: clinical aspects and established and evolving therapies. Lancet 369: 1641–1657. 17499606 10.1016/S0140-6736(07)60751-X

[9] Triantafillidis JK, Merikas E, Georgopoulos F (2011) Current and emerging drugs for the treatment of inflammatory bowel disease. Drug Des Devel Ther 5: 185–210. 10.2147/DDDT.S11290 PMC3084301 21552489

[10] Uhlig HH, Powrie F (2009) Mouse models of intestinal inflammation as tools to understand the pathogenesis of inflammatory bowel disease. Eur J Immunol 39: 2021–2026. 19672896 10.1002/eji.200939602

[11] Olivares-Villagomez D, Mendez-Fernandez YV, Parekh VV, Lalani S, Vincent TL, et al (2008) Thymus leukemia antigen controls intraepithelial lymphocyte function and inflammatory bowel disease. Proc Natl Acad Sci U S A 105: 17931–17936. 19004778 10.1073/pnas.0808242105 PMC2584730

[12] Hayday A, Theodoridis E, Ramsburg E, Shires J (2001) Intraepithelial lymphocytes: exploring the Third Way in immunology. Nat Immunol 2: 997–1003. 11685222 10.1038/ni1101-997

[13] Mucida D, Husain MM, Muroi S, van Wijk F, Shinnakasu R, et al (2013) Transcriptional reprogramming of mature CD4(+) helper T cells generates distinct MHC class II-restricted cytotoxic T lymphocytes. Nat Immunol 14: 281–289. 23334788 10.1038/ni.2523 PMC3581083

[14] Groh V, Steinle A, Bauer S, Spies T (1998) Recognition of stress-induced MHC molecules by intestinal epithelial gammadelta T cells. Science 279: 1737–1740. 9497295 10.1126/science.279.5357.1737

[15] Boismenu R, Havran WL (1994) Modulation of epithelial cell growth by intraepithelial gamma delta T cells. Science 266: 1253–1255. 7973709 10.1126/science.7973709

[16] Komano H, Fujiura Y, Kawaguchi M, Matsumoto S, Hashimoto Y, et al (1995) Homeostatic regulation of intestinal epithelia by intraepithelial gamma delta T cells. Proc Natl Acad Sci U S A 92: 6147–6151. 7597094 10.1073/pnas.92.13.6147 PMC41659

[17] Chen Y, Chou K, Fuchs E, Havran WL, Boismenu R (2002) Protection of the intestinal mucosa by intraepithelial gamma delta T cells. Proc Natl Acad Sci U S A 99: 14338–14343. 12376619 10.1073/pnas.212290499 PMC137885

[18] Cheroutre H, Madakamutil L (2004) Acquired and natural memory T cells join forces at the mucosal front line. Nat Rev Immunol 4: 290–300. 15057787 10.1038/nri1333

[19] Roberts AI, O'Connell SM, Biancone L, Brolin RE, Ebert EC (1993) Spontaneous cytotoxicity of intestinal intraepithelial lymphocytes: clues to the mechanism. Clin Exp Immunol 94: 527–532. 8252812 10.1111/j.1365-2249.1993.tb08229.x PMC1534451

[20] Shires J, Theodoridis E, Hayday AC (2001) Biological insights into TCRgammadelta+ and TCRalphabeta+ intraepithelial lymphocytes provided by serial analysis of gene expression (SAGE). Immunity 15: 419–434. 11567632 10.1016/s1074-7613(01)00192-3

[21] Leishman AJ, Gapin L, Capone M, Palmer E, MacDonald HR, et al (2002) Precursors of functional MHC class I- or class II-restricted CD8alphaalpha(+) T cells are positively selected in the thymus by agonist self-peptides. Immunity 16: 355–364. 11911821 10.1016/s1074-7613(02)00284-4

[22] Yamagata T, Mathis D, Benoist C (2004) Self-reactivity in thymic double-positive cells commits cells to a CD8 alpha alpha lineage with characteristics of innate immune cells. Nat Immunol 5: 597–605. 15133507 10.1038/ni1070

[23] Cheroutre H, Lambolez F, Mucida D (2011) The light and dark sides of intestinal intraepithelial lymphocytes. Nat Rev Immunol 11: 445–456. 21681197 10.1038/nri3007 PMC3140792

[24] Taveirne S, Filtjens J, Van Ammel E, De Colvenaer V, Kerre T, et al (2011) Inhibitory receptors specific for MHC class I educate murine NK cells but not CD8alphaalpha intestinal intraepithelial T lymphocytes. Blood 118: 339–347. 21613250 10.1182/blood-2011-01-331124

[25] Van Den Broeck T, Van Ammel E, Delforche M, Taveirne S, Kerre T, et al (2013) Differential Ly49e expression pathways in resting versus TCR-activated intraepithelial gammadelta T cells. J Immunol 190: 1982–1990. 23338239 10.4049/jimmunol.1200354

[26] de Bruin PA, Crama-Bohbouth G, Verspaget HW, Verheijen JH, Dooijewaard G, et al (1988) Plasminogen activators in the intestine of patients with inflammatory bowel disease. Thromb Haemost 60: 262–266. 3146142

[27] Miseljic S, Galandiuk S, Myers SD, Wittliff JL (1995) Expression of urokinase-type plasminogen activator and plasminogen activator inhibitor in colon disease. J Clin Lab Anal 9: 413–417. 8587011 10.1002/jcla.1860090613

[28] Filtjens J, Taveirne S, Van Acker A, Van Ammel E, Vanhees M, et al (2013) Abundant stage-dependent Ly49E expression by liver NK cells is not essential for their differentiation and function. J Leukoc Biol 93: 699–711. 23475576 10.1189/jlb.0812378

[29] Van Beneden K, De Creus A, Stevenaert F, Debacker V, Plum J, et al (2002) Expression of inhibitory receptors Ly49E and CD94/NKG2 on fetal thymic and adult epidermal TCR V gamma 3 lymphocytes. J Immunol 168: 3295–3302. 11907085 10.4049/jimmunol.168.7.3295

[30] Fraser KP, Gays F, Robinson JH, van Beneden K, Leclercq G, et al (2002) NK cells developing in vitro from fetal mouse progenitors express at least one member of the Ly49 family that is acquired in a time-dependent and stochastic manner independently of CD94 and NKG2. Eur J Immunol 32: 868–878. 11870631 10.1002/1521-4141(200203)32:3<868::AID-IMMU868>3.0.CO;2-A

[31] MacFarlane AWt, Yamazaki T, Fang M, Sigal LJ, Kurosaki T, et al (2008) Enhanced NK-cell development and function in BCAP-deficient mice. Blood 112: 131–140. 18337558 10.1182/blood-2007-08-107847 PMC2435684

[32] Van der Sluis M, De Koning BA, De Bruijn AC, Velcich A, Meijerink JP, et al (2006) Muc2-deficient mice spontaneously develop colitis, indicating that MUC2 is critical for colonic protection. Gastroenterology 131: 117–129. 16831596 10.1053/j.gastro.2006.04.020

[33] Denning TL, Granger SW, Mucida D, Graddy R, Leclercq G, et al (2007) Mouse TCRalphabeta+CD8alphaalpha intraepithelial lymphocytes express genes that down-regulate their antigen reactivity and suppress immune responses. J Immunol 178: 4230–4239. 17371979 10.4049/jimmunol.178.7.4230

[34] Hall LJ, Murphy CT, Quinlan A, Hurley G, Shanahan F, et al (2013) Natural killer cells protect mice from DSS-induced colitis by regulating neutrophil function via the NKG2A receptor. Mucosal Immunol 6: 1016–1026. 23340823 10.1038/mi.2012.140

[35] Kontoyiannis D, Pasparakis M, Pizarro TT, Cominelli F, Kollias G (1999) Impaired on/off regulation of TNF biosynthesis in mice lacking TNF AU-rich elements: implications for joint and gut-associated immunopathologies. Immunity 10: 387–398. 10204494 10.1016/s1074-7613(00)80038-2

[36] Huybers S, Apostolaki M, van der Eerden BC, Kollias G, Naber TH, et al (2008) Murine TNF(DeltaARE) Crohn's disease model displays diminished expression of intestinal Ca2+ transporters. Inflamm Bowel Dis 14: 803–811. 18266230 10.1002/ibd.20385

[37] Meehan TF, Witherden DA, Kim CH, Sendaydiego K, Ye I, et al (2014) Protection against colitis by CD100-dependent modulation of intraepithelial gammadelta T lymphocyte function. Mucosal Immunol 7: 134–142. 23695512 10.1038/mi.2013.32 PMC3795871

[38] Inagaki-Ohara K, Chinen T, Matsuzaki G, Sasaki A, Sakamoto Y, et al (2004) Mucosal T cells bearing TCRgammadelta play a protective role in intestinal inflammation. J Immunol 173: 1390–1398. 15240735 10.4049/jimmunol.173.2.1390

[39] Roselli M, Finamore A, Nuccitelli S, Carnevali P, Brigidi P, et al (2009) Prevention of TNBS-induced colitis by different Lactobacillus and Bifidobacterium strains is associated with an expansion of gammadeltaT and regulatory T cells of intestinal intraepithelial lymphocytes. Inflamm Bowel Dis 15: 1526–1536. 19504616 10.1002/ibd.20961

[40] Jiang W, Wang X, Zeng B, Liu L, Tardivel A, et al (2013) Recognition of gut microbiota by NOD2 is essential for the homeostasis of intestinal intraepithelial lymphocytes. J Exp Med 210: 2465–2476. 24062413 10.1084/jem.20122490 PMC3804938

[41] Hollenbach JA, Ladner MB, Saeteurn K, Taylor KD, Mei L, et al (2009) Susceptibility to Crohn's disease is mediated by KIR2DL2/KIR2DL3 heterozygosity and the HLA-C ligand. Immunogenetics 61: 663–671. 19789864 10.1007/s00251-009-0396-5 PMC2813946

[42] Yadav PK, Chen C, Liu Z (2011) Potential role of NK cells in the pathogenesis of inflammatory bowel disease. J Biomed Biotechnol 2011: 348530. 21687547 10.1155/2011/348530 PMC3114561

[43] Wilson TJ, Jobim M, Jobim LF, Portela P, Salim PH, et al (2010) Study of killer immunoglobulin-like receptor genes and human leukocyte antigens class I ligands in a Caucasian Brazilian population with Crohn's disease and ulcerative colitis. Hum Immunol 71: 293–297. 20036705 10.1016/j.humimm.2009.12.006

[44] Zhang HX, Li JC, Liu ZJ (2008) [Relationship between expression of inhibitory killer cell immunoglobulin-like receptor HLA-Cw ligand and susceptibility to inflammatory bowel disease]. Zhonghua Yi Xue Za Zhi 88: 3108–3111. 19159590

[45] Jones DC, Edgar RS, Ahmad T, Cummings JR, Jewell DP, et al (2006) Killer Ig-like receptor (KIR) genotype and HLA ligand combinations in ulcerative colitis susceptibility. Genes Immun 7: 576–582. 16929347 10.1038/sj.gene.6364333

[46] Yokoyama WM, Plougastel BF (2003) Immune functions encoded by the natural killer gene complex. Nat Rev Immunol 3: 304–316. 12669021 10.1038/nri1055

[47] Yusa S, Catina TL, Campbell KS (2002) SHP-1- and phosphotyrosine-independent inhibitory signaling by a killer cell Ig-like receptor cytoplasmic domain in human NK cells. J Immunol 168: 5047–5057. 11994457 10.4049/jimmunol.168.10.5047

[48] Campbell KS, Purdy AK (2011) Structure/function of human killer cell immunoglobulin-like receptors: lessons from polymorphisms, evolution, crystal structures and mutations. Immunology 132: 315–325. 21214544 10.1111/j.1365-2567.2010.03398.x PMC3044898

[49] Apostolaki M, Manoloukos M, Roulis M, Wurbel MA, Muller W, et al (2008) Role of beta7 integrin and the chemokine/chemokine receptor pair CCL25/CCR9 in modeled TNF-dependent Crohn's disease. Gastroenterology 134: 2025–2035. 18439426 10.1053/j.gastro.2008.02.085

