# Correction: Ly49E Expression on CD8αα-Expressing Intestinal Intraepithelial Lymphocytes Plays No Detectable Role in the Development and Progression of Experimentally Induced Inflammatory Bowel Diseases

**DOI:** 10.1371/journal.pone.0124464

**Published:** 2015-04-09

**Authors:** The PLOS ONE Staff

Dr. Els Louagie is not included in the author byline. She should be listed as the seventh author and affiliated with Department of Rheumatology, Ghent University, Ghent, Belgium. The contributions of this author are as follows: Performed the experiments, analyzed the data, and contributed reagents/materials/analysis tools.

The Funding section should read: This work was supported by grants from the Foundation against Cancer, a foundation of public interest (2010–166) (http://www.kanker.be/grants) (G.L.), by the Fund for Scientific Research (FWO) (G.0187.13) (http://www.fwo.be/en/fellowships-funding/research-projects/research-project/) (G.L.) and by the BOF of Ghent University (BOF11/GOA/005) (http://www.ugent.be/nl/onderzoek/financiering/bof/goa/overzicht.htm) (J.P, G.L., B.V, T.T.). A.V.A, J.F., M.V. are supported by the Institute for the Promotion of Innovation through Science and Technology Flanders (IWT-Vlaanderen) (http://www.iwt.be/subsidies/sb). S.T. is supported by the BOF of Ghent University. T.K. is supported by the FWO. E.L. is supported by the Interuniversity Attraction Poles Programme of the Belgian Science Policy Office (IAP project DevRepair). The funders had no role in study design, data collection and analysis, decision to publish, or preparation of the manuscript.
